# ﻿*Orobancheingens* (Orobanchaceae) – a poorly known species of the Greater Caucasus: taxonomic problems, distribution, hosts and habitats

**DOI:** 10.3897/phytokeys.193.79886

**Published:** 2022-03-17

**Authors:** Renata Piwowarczyk, Óscar Sánchez Pedraja, Alexander V. Fateryga, Sergey A. Svirin

**Affiliations:** 1 Center for Research and Conservation of Biodiversity, Department of Environmental Biology, Institute of Biology, Jan Kochanowski University, Uniwersytecka 7, PL-25-406 Kielce, Poland Jan Kochanowski University Kielce Poland; 2 Grupo Botánico Cantábrico, ES-39722 Liérganes (Cantabria), Spain Grupo Botánico Cantábrico Liérganes Spain; 3 T.I. Vyazemsky Karadag Scientific Station, Nature Reserve of the Russian Academy of Sciences, Branch of A.O. Kovalevsky Institute of Biology of the Southern Seas, Nauki Str. 24, Kurortnoye, Feodosiya 298188, Russia Nature Reserve of the Russian Academy of Sciences, Branch of A.O. Kovalevsky Institute of Biology of the Southern Seas Feodosiya Russia; 4 Urban Development Institute, Sevastopol State University, Kornilova Emb. 1, Sevastopol 299011, Russia Sevastopol State University Sevastopol Russia; 5 Nikitsky Botanical Garden – National Scientific Center, Nikita, Yalta 298648, Russia Nikitsky Botanical Garden – National Scientific Center Yalta Russia

**Keywords:** Caucasus, distribution range, *Heracleum*, holoparasitic Orobanchaceae, host, *Orobanche*, typification clarification

## Abstract

*Orobancheingens* is an endemic species from the Caucasus, especially the Greater Caucasus, parasitising on large Apiaceae (usually *Heracleum*). This species was misclassified over the years and little was known about its range and habitats. Here, we clarify the typification, as well as provide notes about the taxonomy of this species. Additionally, we presented distribution, habit and host range of *O.ingens* and morphological features that distinguish it from similar species.

## ﻿Introduction

This taxon was described by Beck (1922) on the basis of a part of the materials collected by Th. Alexeenko in Kasi-Kumukh (Dagestan). These materials are preserved on three herbarium sheets in LE. These three sheets contain original labels, two of them equal and printed in blue ink by Alexeenko (LE01015385 and LE01015386) and the third handwritten, probably by Beck (LE01015387); furthermore, the three labels have two of Beck’s manuscript numbers: 10524 (LE01015386) and 10544 (LE01015385 and LE01015387). Beck considered the most typical material (today with the barcode LE01015386) as a robust form (f. ingens) of *O.alba* Stephan ex Willd.; remaining specimens, collected at the same place and on the same date (today with the barcodes LE01015385 and LE01015387) and with the number 10544 on their main labels, were determined by him as f. bidentata of *O.alba*. Beck seemed to see transitional forms between the specimens of Alexeenko’s gathering, while in our opinion, they are all similar and correspond to the same species (*O.ingens* (Beck) Tzvelev). Later, Beck (1930) included his f. ingens within the taxonomically elevated var. bidentata [O.albavar.bidentataf.ingens Beck], presumably based on the morphology of the calyx segments (deep and long bidentate). Novopokrovsky, in 1947, in his herbaria reviews (‘Notae criticae’), considered this species closely related to *O.crenata* Forssk.; on a herbarium label, he even came to subordinate the form described by Beck to *O.crenata*.

Years later, Tzvelev (1957 and 1958 [in Novopokrovsky and Tzvelev 1958]), supposedly based on the material deposited in LE and collected in the Greater Caucasus (cf., for example, LE s.n. – Blyumental and Karpova 1946 n. 139), published O.alsaticavar.heracleiTzvelev. Tzvelev did not directly or indirectly mention f. ingens described by Beck and, furthermore, indicated that his new variety can be found in Azerbaijan extending its distribution range to the Greater Caucasus. Finally, on the basis of the same gathering of Alexeenko used by Beck (1922), Tzvelev (1990) raised the taxon described by Beck (1922) to the rank of species, indicating its correct basionym, but continued to point out its relationship with the *O.alsatica* aggregate.

Tzvelev (1990: 182; 2015: 211) indicated the sheet of Th. Alexeenko (LE s.n.; nowadays as LE01015386) as the ‘Typus’ of f. ingens with the reference to the number ‘10524’ (today in the scan of the LE herbarium covered by a detached flower). This sheet (with a single uniform gathering) is the lectotype (Turland et al. 2018: Art. 9.3). Although Beck did not indicate a single sheet, of the three sheets collected by Alexeenko from this location and available at present in LE, only one was used by Beck to describe his new form (Turland et al. 2018: Art. 9.4, ‘original material’). This sheet, on the original label of Alexeenko, has, in the manuscript by Beck, in black ink, the number ‘10524’ and the annotation ‘*Heracleum* parasitica’. In addition, it contains another label manuscript by Beck, the description of his new form. This description is very similar to that published by Beck (1922, for example, ‘Floribus ... 27–28 mm longi’), almost literally copied by himself in 1930. The other two sheets (LE01015385 and LE01015387), which contain Tzvelev’s (1988) review label as ‘Isotypus’, are not really isotypes because they are not a part of the original material used by Beck (cf. the LE virtual herbarium as ‘Isotype of *Orobancheingens* (G. Beck) Tzvelev’), who, at the time of describing his f. ingens, treated them to belong to a different taxon of the same rank, f. bidentata.

Moreover, the new variety (var. heraclei), described by Tzvelev (1957) and (1958) [in Novopokrovsky and Tzvelev 1958], was not published validly according to the ICN (Turland et al. 2018). The first time (Tzvelev 1957), it was published with a Russian, but no Latin description or diagnosis, making it a not validly published name (Turland et al. 2018: Art. 39.1). For the second time of publication (Tzvelev 1958) [in Novopokrovsky and Tzvelev 1958], indicated by Tzvelev (2015) as ‘nom. nud.’, the same applies: the name, accompanied by some diagnostic characters, the habitat and the host plant are described, but everything is in Russian without a Latin description or diagnosis.

## ﻿Clarification and history of the typification

### ﻿ Orobanche ingens

Taxon classificationPlantaeLamialesOrobanchaceae

(Beck) Tzvelev in Novosti Sist. Vyssh. Rast. 27: 182 (1990)

B482DFB6-B2D0-51BC-B35B-5ED9D326D53D

Figs 1 , 2

Orobanche alba f. ingens Beck in Repert. Spec. Nov. Regni Veg. 18: 38[470] (1922)

#### Type.

**Figure 1.**
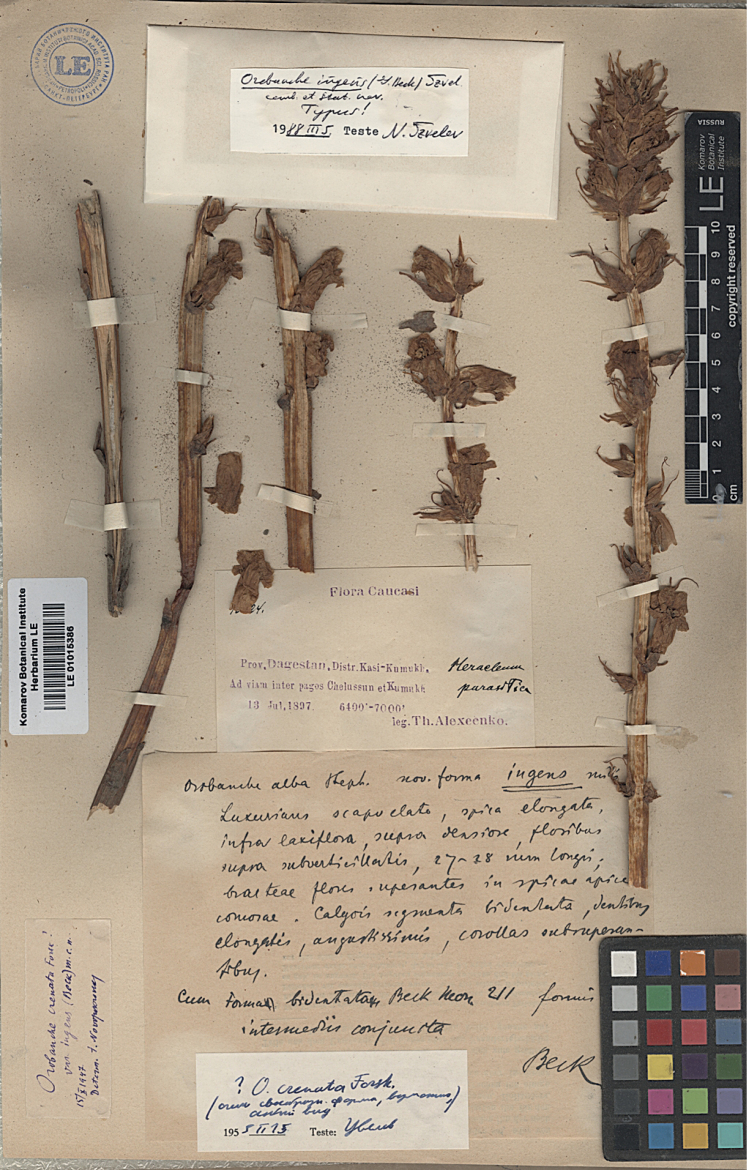
Lectotype of *Orobancheingens* (LE01015386) (http://re.herbariumle.ru/01015386).

lectotype (Tzvelev 1990: 182; Turland et al. 2018: Art. 9.3, 7.11; we do not know if additional elements were used by Beck): Russia: 1. “Flora Caucasi / 10524 [m. Beck]. / *Heracleum* parasitica [m. Beck] / Prov. Daghestan, Distr. Kasi-Kumukh [Kazi-Kumykh, Laksky distr.]. / Ad viam inter pagos Chelussun et Kumukh / 13 Jul 1897 6400´–7000´ / leg. *Th. Alexeenko*”. – 2. “Orobanche alba Steph. nov. forma ingens mihi / Luxurians… [description] … / Cum forma bidentata Beck Mon. 211 formis / intermediis conjunta / Beck [m. Beck]”. – 3. “Orobanche crenata Forssk.! / var. ingens (Beck) m.c.n. / 15.X.1947 / Determ. I. Novopokrovsky”. – 4. “? *O.crenata* Forssk. / … / 1955.II.15 Teste: Tzvelev”. – 5. “*Orobancheingens* (G. Beck) Tzvel / comb. et stat. nov. / Typus! / 1988.III.5 Teste Tzvelev” (LE01015386, Fig. 1).

#### Additional non-type material.

**Figure 2.**
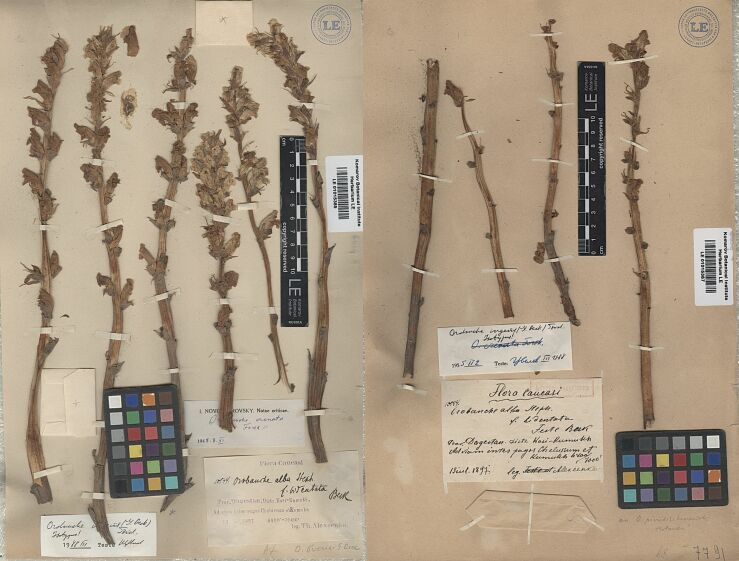
Additional non-type material of *Orobancheingens* (LE01015385, LE01015387) (http://re.herbariumle.ru/01015385, http://re.herbariumle.ru/01015387).

Russia: 1. “Flora Caucasi // 10544. OrobanchealbaSteph. /f.bidentata Beck [m. Beck] // Prov. Daghestan, Distr. Kasi-Kumukh [Kazi-Kumykh, Laksky distr.]. / Ad viam inter pagos Chelussun et Kumukh / 13 Jul 1897 6400´–7000´ / leg. *Th. Alexeenko*”. – 2. “Af. *O.owerini* G. Beck [m. Beck, handwritten on the sheet]”. – 3. “I. Novopokrovsky. Notae criticae / *Orobanchecrenata* / Forssk.! / 1948.8.XI”. – 4. “*Orobancheingens* (G. Beck) Tzvel / Isotypus! / 1988.III Teste Tzvelev” (LE01015385, Fig. 2).

1. “Flora Caucasi / 10544. / OrobanchealbaSteph. /f.bidentata Beck / Prov. Daghestan, Distr. Kasi-Kumukh [Kazi-Kumykh, Laksky distr.]. / Ad viam inter pagos Chelussum [sic] et Kumukh / 6400´–7000´ / 13 Jul 1897 / leg. *Th. Alexeenko* [m. Beck]”. – 2. “an. *O.picridis*-*hieraciodes* / Holandre [handwritten on the sheet]”. – 3. “*Orobancheingens* (G. Beck) Tzvel / Isotypus! / *O.crenata* Forsk. / 1955.II.2 [date of the first revision] Teste Tzvelev III.1988” (LE01015387, Fig. 2).

#### Synonyms.

Orobanchealsaticavar.heraclei Tzvelev, Fl. Azerb. 7: 591 (1957), nom. inval. (Turland et al. 2018: Art. 39.1); Orobanchealsaticavar.heraclei Tzvelev in Schischk., Fl. SSSR 23: 111 (1958), nom. inval. (Tzvelev 2015: 211, nom. nud. [sic]).

#### Misapplied names.

Orobanchealbavar.bidentata sensu Beck in Engl., Pflanzenr. 96: 155 (1930) [saltem p.p.], non O.albaf.bidentata Beck in Biblioth. Bot. 19: 211 (1890).

##### ﻿Distribution, ecology and taxonomic problems

**Figure 3.**
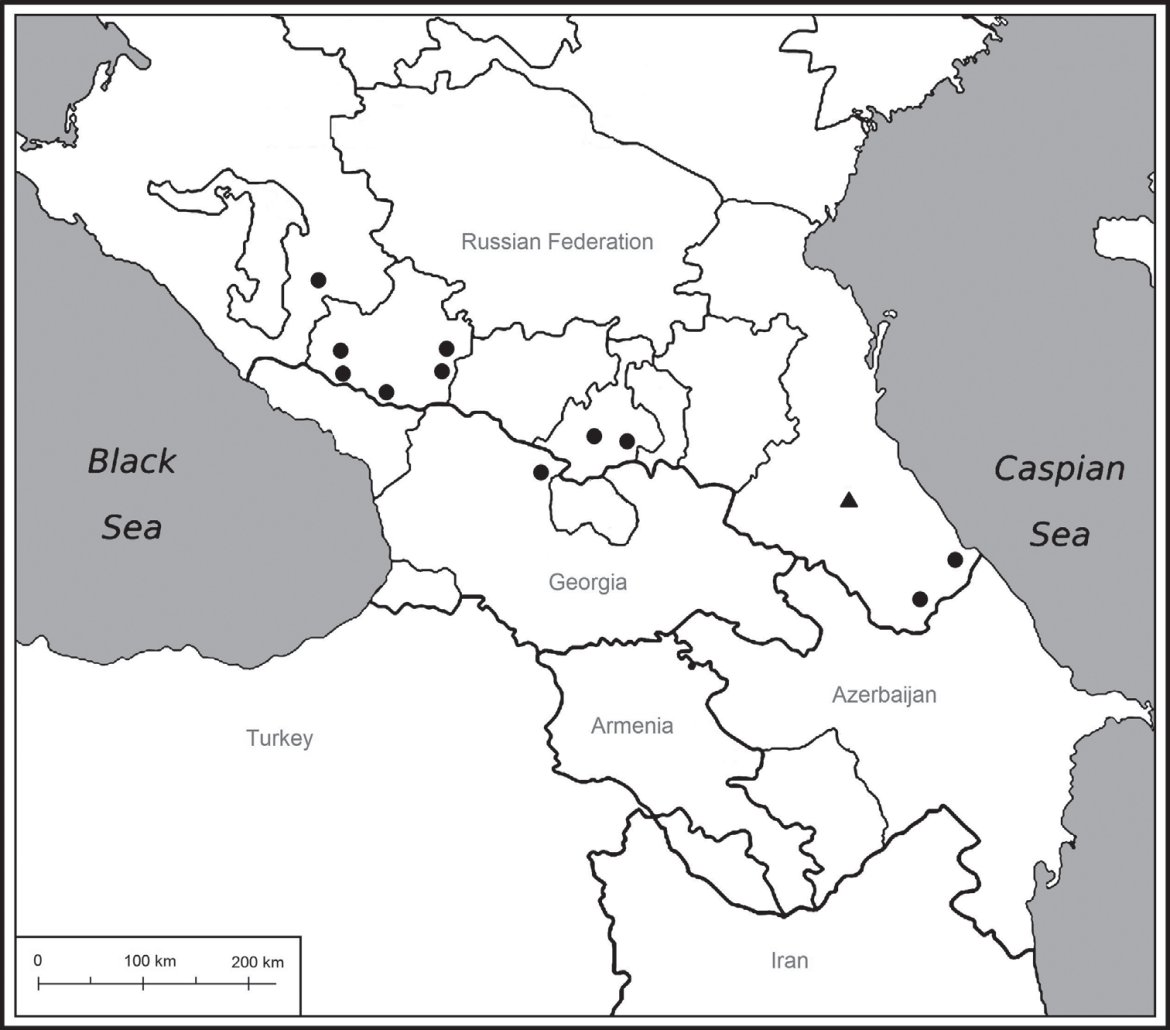
Distribution of *Orobancheingens*, triangle indicates *locus classicus*.

**General distribution.** Caucasus, mainly Greater Caucasus range, Russia (Krasnodar Krai, Karachay-Cherkessia, North Ossetia, Dagestan) and Georgia. Needs confirmation in Azerbaijan. Endemic to the Caucasus (Fig. 3).

**Specimens examined.** Georgia. Racha-Lechkhumi and Kvemo Svaneti prov., tall herb communities, forest edges and glade, near the trail to Udziro lake, 1.5–2 km S of Shovi Village, 42°41'07"N, 43°39'56"E, 2050–2100 m a.s.l., on *Heracleumleskovii*, but probably also on *Ligusticumalatum*, 18 July 2018, *R. Piwowarczyk* (KTC); Russia: Dagestan. Distr. Kasi-Kumukh [Laksky distr.], ad viam inter pagos Chelussun et Kumukh, 6400´–7000´, 13 July 1897, *Th. Alexeenko* (LE) [as *O.ingens* by Tzvelev in 1988, Typus]; prov. Dagestan, distr. Samur, ad riparian fl. Dulty-chaj, 7300´, 12 July 1897, *Th. Alexeenko* (LE) [as “10526” O.albaf.rubiginosa by Beck (Beck 1930: 148, sub *O.alba*), *O.alsatica* by Novopokrovsky in 1949, *O.flava* by Tzvelev in 1955, *O.ingens* by Tzvelev in 1988]; Dagestan, Dokuzparinskiy distr., Kiler, meadow near road, parasitising on *Heracleumsosnowskyi* [root attachment verified], 41°22'50"N, 47°53'55"E, [ca. 1130 m a.s.l.], 26 June 2021, *A.V. Fateryga & S.A. Svirin* (KTC, YALT); Karachay-Cherkessia. North Caucasus, upper reaches of the Bolshaya Laba river, upper part of Zagedanka river, tall grassy vegetation in subalpine meadow, on the roots of *Heracleum*, 2300 m a.s.l., 19 July 1946, *Blyumental & Karpova* (LE) [as *O.owerinii* by Novopokrovsky in 1946, O.alsaticaKirschl.var.heraclei Tzvel. by Tzvelev in 1955, *O.ingens* (G. Beck) Tzvel. by Tzvelev in 1988]; North Caucasus, basin of the Urup river [Kuban distr.], 9 Aug 1945, *V.I. Grubov & L.I. Ivanina* (LE) [as *O.ingens* by Tzvelev in 1988]; near Dombay, Alibek river valley, 29 July 2014, *M. Skotnikova* [phot., https://www.plantarium.ru/page/image/id/268327.html, https://www.plantarium.ru/page/image/id/256514.html, https://www.plantarium.ru/page/image/id/256512.html, as *O.alsatica*]; Dombay, Alibek river valley, 26 August 2013, *S. Banketov* [phot., https://www.plantarium.ru/page/image/id/205049.html, https://www.plantarium.ru/page/image/id/205050.html, https://www.plantarium.ru/page/image/id/205051.html, https://www.plantarium.ru/page/image/id/205052.html, as *O.alsatica*]; Karachay-Cherkessia, Dombay, Dombay-Ulgen river valley, above the Russian glade, about 2000 m a.s.l., 21 July 2010, *E. Komarov* [phot., https://www.plantarium.ru/page/image/id/66557.html, https://www.plantarium.ru/page/image/id/66558.html]; Karachayevsky distr., near Dombay, 11 Aug 2010, *E. Suslova* [phot., https://www.inaturalist.org/observations/70214391, as *O.alsatica*]; Karachay-Cherkessia, Malokarachaevsky distr., slope of Bolshoi Bermamyt peak, ca. 2000 m a.s.l., subalpine meadow, 5 Aug 2017, *T. Gaidash* [phot., https://www.plantarium.ru/page/image/id/527245.html, https://www.plantarium.ru/page/image/id/527246.html, as *O.grossheimii*]; Karachay-Cherkessia, Malokarachaevsky distr., Eshkakon river valley, 15 June 2013, *I. Tabunova* [phot., https://www.plantarium.ru/page/image/id/193231.html]; Krasnodar Krai. North Caucasus, Krasnodar territory, Mostovskoi distr., 6–7 km N from Shedok, vicinity of Dyatlovo, small piece of fallow land at the field margin, not far from dried-up stream, on *Heracleummantegazzianum*, 4 June 2002, *D.V. Geltman* (LE) [as *O.alsatica*]; North Ossetia. Vladikavkaz, July 1888, *Akinfiew* (LE) [as *O.flava*? by Tzvelev in 1955, as *O.ingens* by Tzvelev in 1988]; North Ossetia, Irafsky distr., upper part of Urukh river, ca. 1700 m a.s.l., subalpine meadow, 9 July 2016, *M. Skotnikova* [phot., https://www.plantarium.ru/page/image/id/479013.html, as *O.alsatica*].

**Habitat.** In tall herbaceous habitats, mainly in subalpine meadows, pastures, edges of forests and shrubs, forest glades, near river valleys, as well as fallow lands, usually (1000) 2000–2300 (2500) m a.s.l.

In Georgia, in the locality on a subalpine glade, numerous pollinators of this species – workers of *Dolichovespulasylvestris* (Scop.) (Hymenoptera, Vespidae) – have been observed.

**Host.** Parasitises *Heracleum* species (Apiaceae), such as *H.leskovii* Grossh., *H.sosnowskyi* Manden. and *H.mantegazzianum* Sommier & Levier. Probably also on other large Apiaceae species, such as *Ligusticumalatum* (M. Bieb.) Spreng. (= *Cnidiocarpaalata* (M. Bieb.) Pimenov & Kljuykov), but this needs confirmation (observed by R. Piwowarczyk in Georgia, but root attachment was not verified).

Apart from *O.ingens*, only one *Heracleum* parasite is known – *Phelipanchesevanensis* Piwow., Ó. Sánchez & Moreno Mor., described from the slopes of Sevan lake in Armenia, where it parasitises *H.trachyloma* Fischer & Meyer (Piwowarczyk et al. 2017, 2019).

**Phenology.** Flowering (June) July–August, fruiting (July) August (September).

**Figure 4.**
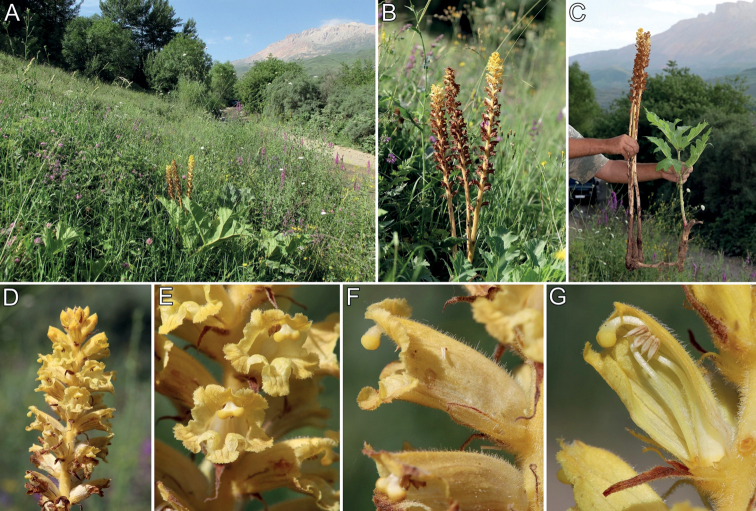
*Orobancheingens* from Kiler in Dagestan **A, B** habitat and general habit **C** attachment with host root of *Heracleumsosnowskyi***D** top of inflorescence **E** flowers in front view **F** flower in lateral view **G** flower in longitudinal-section. Phot. A. Fateryga.

**Note.** At first, the species was mistakenly described as a form of *O.alba* (subsect. Glandulosae); next Tzvelev (1990, 2015) continued to indicate the relationship of *O.ingens* with *O.alsatica* aggr., especially with *O.bartlingii* Griseb. (subsect. Curvatae). However, according to morphological features (Figs 1, 2, 4, Table 1), it is far from the *O.alsatica* group and subsect. Curvatae, but belongs to the *Minores/Speciosae* subsection. In the *Minores/Speciosae* subsection, the corolla is usually tubular or campanulate, almost straight in the middle part, the pubescence of the corolla is usually a mixture of longer and stiff whitish hairs, the calyx segments are long, entire or bidentate, usually subulate or filiform at the tip. In contrast, in the subsect. Curvatae, the corolla is usually tubular-infundibuliform or ± broadly tubular, the dorsal line of the corolla is evenly curved over its entire length, the pubescence is rarely a mixture of whitish hairs. Molecular studies do not indicate the validity of dividing the species into the subsect. Speciosae because the species included here are both morphologically and genetically very similar to those of the subsect. Minores (Piwowarczyk et al. 2021). It is worth emphasising that individuals of *O.ingens* are usually very tall, over 60 to 100 cm, with corolla (20–) 23–24 (–30) cm long, usually with a mixture of longer and stiff whitish hairs (Fig. 4F; without dark glandular hairs which are characteristic for the subsect. Glandulosae) and both the corolla and stem can be very variable in colour, even within one population, from yellow to pink and purple; additionally, the colour of the stigma can be variable, from yellow through to orange, pink to purple (Fig. 4 and photos from Plantarium.ru and iNaturalist.org, links above).

**Table 1.** Distinctive morphological characters and hosts of the studied species and its Caucasian relatives.

	*O.bartlingii*	*O.ingens*	*O.owerinii*	*O.laxissima*	*O.minor*
**Inflorescence**	cylindrical to ovate, ± dense, usually shorter than the rest of the stem	usually long, rarely short (small specimens), cylindrical, somewhat lax, usually many-flowered	usually short-cylindrical to ovate, ± lax, usually few-flowered	usually long, or less frequently short (small specimens), cylindrical, very lax, usually many-flowered	usually long, rarely short (small specimens), cylindrical, dense at the first of anthesis, becoming lax later, usually many-flowered (except small specimens)
**Floral bract**	about two thirds of the length of the corolla	as long as the corolla tube, slightly shorter than the corolla	as long as the corolla or slightly shorter or longer than the corolla	as long as the corolla tube, slightly shorter than the corolla	as long as the corolla
**Calyx segments**	bidentate, teeth short, usually subulate at tip	deeply bidentate, teeth frequently filiform	segments entire or less frequently bidentate, narrowly subulate	usually entire or less frequently bidentate, rarely with 4 teeth, teeth narrowly subulate to filiform	bidentate or less frequently entire, teeth subulate to narrowly subulate but not filiform
**Corolla, length**	12‒18 mm	(20–) 23–24 (–30) mm	15–20 (‒30) mm	(20) 22–24 (–25) mm	10–19 mm
**Corolla, form**	tubular	campanulate	campanulate-infundibuliform	tubular-infudibuliform	tubular
**Corolla, dorsal line**	evenly curved at the proximal and distal part, almost straight at the middle	almost straight, except evenly curved at the proximal part	almost straight, except evenly curved at the proximal part and, sometimes, slightly bent forward at the distal part	almost straight, except evenly curved at the proximal part and, sometimes, slightly bent forward at the distal part	evenly curved at the proximal part, almost straight at the middle and slightly bent forward at the distal part
**Corolla, colour**	yellow, brownish-yellow, pinkish ± tinged with purple	yellow, rarely violet, dark pink	pale violet to purple, rarely cream	purple, dark or light pink, rarely dirty yellow, light brown, sometimes with ± dark purplish veins	whitish or yellowish-white, more or less coloured purple, with dark purplish veins, rarely entirely yellow
**Filaments**	inserted 1–3 mm above the corolla base; with long hairs at the basal half and sparsely glandular pubescent under the anthers	inserted 1 (–2) mm above the corolla base; with long hairs at the basal half and sparsely glandular pubescent (almost glabrous) under the anther	inserted 3–4 mm above the corolla base; hairy at the basal half (or third) and sparsely glandular pubescent under the anthers	inserted (2–)3–5 mm above the corolla base; hairy at the base and sparsely glandular pubescent under the anthers	inserted 2–3 (–5) mm above the corolla base, glabrous or sparsely hairy at the base and sparsely glandular pubescent under the anthers
**Stigma**	yellow	mainly yellow or orange, rarely reddish, violet, dark pink	reddish to deep purple	dark pink, purple, violet, rarely orange, pale to dark yellow	pinkish, ± deep purple, rarely yellow or whitish
**Host**	Apiaceae (*Seseli*)	Apiaceae (*Heracleum*)	Fabaceae (*Trifolium* and *Vicia*) and rarely Asteraceae (*Leontodon* and *Lactuca*)	trees and shrubs, Betulaceae, Oleaceae, Fagaceae, Sapindaceae, Cornaceae, Lythraceae, Fabaceae, Anacardiaceae (e.g., *Fraxinus*, *Fagus*, *Carpinus*, *Rhus*)	Fabaceae, Asteraceae, Apiaceae, etc. (e.g., *Trifolium*, *Medicago*, *Hypochaeris*, *Leontodon*, *Chondrilla*, *Daucus*)

## Supplementary Material

XML Treatment for Orobanche ingens
